# Memoir of the Life and Writings of the Late Dr. Armstrong

**Published:** 1833-04-01

**Authors:** 


MEMOIR OF THE LIFE AND MEDICAL OPINIONS OF JOHN ARMSTRONG, M.D., TO WHICH IS ADDED AN INQUIRY INTO THE FACTS CONNECTED WITH THOSE FORMS OF FEVER ATTRIBUTED TO MALARIA, OR MARSH EFFLUVIUM. By *Francis Boott*, M.D. Volume the first, pp. 616, with a profile of Dr. Armstrong. Baldwin, Feb. 1833.

THE biographer was a favourite pupil, and latterly co-lecturer with Dr. Armstrong, having enjoyed the confidence and the friendship of that distinguished physician for the last ten years of his life. Dr. Boott must therefore be well qualified (as far as intimate knowledge of the deceased goes) for the task which he has undertaken, though some acquaintance with the illustrious dead, and a careful perusal of the memoir before us, have induced a suspicion that friendship, we might say enthusiastic admiration of the talents of his preceptor, has concealed from the biographer's view every foible which Dr. Armstrong, in common with humanity, must have possessed, however small in number or trifling in kind. This, indeed, is an amiable failing on the part of the biographer; but we suspect that the portrait would have been nearer to nature, and therefore more valuable, had the shades of weakness been skillfully introduced among the prominent features of strength. Dr. Armstrong had such a preponderance of virtues, talents, and acquirements, that the shades in question would have been a relief to the contemplative eye, without being of the slighest disadvantage to the fame or character of the deceased. When a biographer expressly declares that "he knows of no defects in the moral nature" of the deceased, our inference must inevitably be, that the biographer has been a superficial or a partial observer;—since no man ever yet existed on this earth—(with one exception)—who had no "defects in his moral nature." But more of this hereafter. We shall now give a condensed outline of Dr. Armstrong's life.

Dr. A. was born at Ayres Quay, Bishopwearmouth, in May, 1784. His parents were of humble origin—his father having received no education, and being manager of a glass-work—but though untaught, he was possessed of superior natural abilities. To his mother, a Miss Robson, Dr. A. is supposed to be indebted for the advantages of education, that amiable woman having exerted her slender means for his support during the period of studies, both at school and at the University. The youth had been put to school at a very early age, but at the expiration of some years, it was found that he had not acquired the most simple elements of learning. He was then removed to another school, where he was at first, "considered by his friends as incapable of learning anything!" This, however, must have been owing to mismanagement, for he soon made good progress under a new tutorship. He acquired confidence in himself, and evinced the laudable ambition of even excelling his competitors in erudition. He left the school at the age of 16 years, and was apprenticed to an apothecary, Mr. Watson, at Monkwearmouth; but soon took a dislike to the profession, and returned home, where he led a desultory sort of life for two or three years. An exertion was then made by his humble parents, and the youth was sent to Edinburgh, where he was obliged to use the most rigid economy. During the desultory years above-mentioned, it appears that Dr. A. formed several visionary schemes for his future life, one of which was, to go to London and try his fortune on the wide but perilous ocean of literature.

"He early showed a taste for poetical composition, and wrote several fugitive pieces which attracted the notice of his companions. He frequently mentioned to me that, while he was uncertain of his future career, he meditated writing a tragedy on the story of Boethius, whose character and fate, as recorded by Gibbon, had made a strong impression on his mind, and that he had entered so far upon this ambitious attempt as to sketch out a plan of the work, and an outline of the principal characters. This idea I think never entirely deserted him, for he often spoke of it, and seemed to wish for some relaxation from the anxious duties of his profession, that he might indulge in literary pursuits." 5.

Dr. A. graduated in June, 1807, and settled the same year at Sunderland. Here he had the stimulus of necessity to sharpen his intellects and quicken his industry. A circumstance here occurred which proved very fortunate for the young physician. There was a wealthy invalid in Sunderland, with whose brother Dr. A. had become acquainted at Edinburgh. He had chronic dysentery or diarrhœa for more than two years, which had baffled the skill of all the medical men of the place. Being fresh from the prelections of Hamilton, Dr. A. had recourse to purgatives, by which the complaint was cured. The praises of the youthful Doctor were for ever on the tongue of the grateful patient—and there is no doubt but this proved an extremely happy hit of the young aspirant for professional fame.

During the ten years that he practised in Sunderland, he contributed several papers to the Edinburgh Medical and Surgical Journal—especially on Brain Fever from Intoxication—on Puerperal Fever, &c. In 1813 he published "Facts and Observations on Puerperal Fever," a work which first brought him into notice, and to which he bore the love of a parent till the latest day of his existence. His mind was now drawn particularly to the subject of fever, and in 1816 his celebrated work on typhus appeared, and raised him, as it were, per saltum, to a high eminence in his profession. We believe we were the first to give a public expression of approbation to the work, for on the first of January, 1817, we commenced a series of reviews of it, which tended not a little to excite the attention of the faculty to the work reviewed, on both sides of the Atlantic. One short quotation will shew the manner in which we spoke of Dr. Armstrong's book. "It stamps the reputation of its author with a character so indelible, that TIME, which soon washes away all subordinate impressions, will only render its features more conspicuous."*

Dr. A. wrote, in grateful terms, to the Editor of this Journal, and an epistolary correspondence, followed by personal intimacy, was the result. We know, both from oral and literal communications, that the review in question first suggested the idea of removing from the confined and narrow scene where Dr. A. then practised, to the boundless theatre of the metropolis. This idea was strengthened by the Editor's announcing to Dr. Armstrong, his own intention of abandoning provincial practice, and of settling in London. In fact, these two gentlemen took up their residence in the metropolis within six months of each other, Dr. A. having the start of Dr. Johnson.

In February, 1818, Dr. A. took apartments in Great James Street, Bedford Row, a most gloomy street for a young physician to settle in, on first coming to the great vortex. We are quite certain that much of the despondency of mind which Dr. A. was subject to during the first year or two was occasioned by the gloominess of the street.

"All those domestic sympathies upon which he so much depended for happiness were far removed from him, and he felt as it were alone in the world, anxious about his present and uncertain of his future fortunes. He has often told me that the loneliness of his situation at times overpowered him; and that so oppressive was the busy scene around him in which he stood a stranger, uncared for and unknown, that he sometimes found relief in tears, and tried to drown the consciousness of sorrow, by seeking sleep in his darkened chamber at noon." 22.

It was during this period that we became intimately acquainted with Dr.

* Medico-Chirurgical Journal, January, 1817. The Edinburgh Journal only devoted four or five pages to the examination of Dr. A.'s work.

A. there being, unfortunately, but too much leisure-time on hand, giving occasion to almost daily reflections on the past, the present, and the future. A few feeless days would give rise to the most lugubrious prophecies, while a little sunshine would change all again to sanguine anticipation.

It was in the Summer of this year that Dr. Armstrong was rejected at the College of Physicians, an event that formed an epoch in his life, and, we are quite confident, accelerated his death. It seemed to precipitate him into ruin, by disqualifying him for the office of physician to the Fever Hospital, for which he was a candidate—and by proclaiming to the world that his book was an imposture and himself an ignoramus. But the event was just the reverse of what himself, his friends, and even his enemies (if he then had any) anticipated. The *re-action* raised him up numerous advocates in the profession—and even in the managing committee of the Fever Hospital, the standing law of the institution was suspended, and he was elected physician in the teeth of the Warwick-lane rejection. But these were minor consequences. The wound which his feelings suffered by this insult, festered till the latest day of his existence. Its smart proved a constant excitement and stimulus to industry, prompting him to almost superhuman exertion, in order to wipe off the disgrace and revenge the affront. Every one who has been personally acquainted with Dr. A. or who has heard his lectures, must remember the tone of indignation with which he always spoke of the medical institutions of this country.

He owed his success, in the opinion of his biographer, to two causes.

"Those members of the general profession who had once experienced the benefit of his counsel and assistance, could seldom be induced to recommend any other physician, so strongly impressed were they with the simplicity, the originality and success of his views and practice: and those families who had once had an opportunity of feeling the effects of the gentleness and delicacy of his manner, could think of no other adviser." 33.

We now find him physician to the Fever Hospital, and residing with his family in Southampton Row. Every one supposed he was rising fast into practice and reputation; but the truth was, that his finances were becoming exhausted, and he was a prey to the most dreadful anxiety. This we know from personal acquaintance, and it is corroborated by Dr. Boott.

"An anecdote, connected with the period of his greatest difficulties, in the year 1820, when his funds were nearly exhausted, is too honourable to himself and to the late Mrs. Oliphant, of Gask in Scotland, to be omitted.

He had naturally been led to take an unfavourable view of his prospects, from the prejudices excited against him by the conduct of the College of Physicians, and from an experience of the unavoidable expense attending a physician's opening career in London, which appeared the more formidable to him, from the injury that he imagined had been done to his reputation. His mind was at one time so much a prey to anxiety, that he entertained serious thoughts of removing from town. This idea was communicated to Mrs. Oliphant, in whose family he had practised for several years, and to whom his worth was fully known. She immediately remonstrated against it. With a delicacy which added to the extent of her kindness, she advised him to set up his carriage, and insisted that he should draw upon her banker for any sums he might require, till his income should prove equal to his wants. This noble act of devotion to a pure and exalted friendship, was honoured as it deserved to be; for Dr. Armstrong availed himself of the liberal offer, and the fruits of this beautiful instance of mutual confidence were to remove at once the apprehensions he laboured under, and to fortify his mind with a confiding hope of ultimate success. He never spoke of this disinterested act of friendship without emotion, and he always attributed his subsequent prosperity to it, as it reconciled him to the difficulties he had to encounter, and enabled him to employ his mind, unfettered by anxiety to the discharge of the responsible duties of his situation as Physician to the Fever Hospital." 62.

This anecdote may prove valuable to some youthful aspirants for metropolitan fame and fortune, who may think that Dr. Armstrong's talents and writings secured him success from the moment he appeared on the great theatre of London. The fact is, that had it not been for the timely succour of Mrs. Oliphant, Dr. A. would have been obliged, in all probability, to quit the metropolis, and end his days in some obscure provincial town. We know of more than one, two or three meritorious individuals, who, having acquired some notice by their writings, have ventured into the whirlpool of Modern Babylon, with the sanguine hope of soon rising into eminence—too soon, alas! to be awakened from their golden dreams by disappointment and poverty!

Before quitting this part of the subject, we may here state our belief, that Dr. A.'s rejection at the College of Physicians was not owing to any hostility on the part of the censors, but to the antiquated and ridiculous system or mode of testing a medical man's acquirements by a string of questions in a dead language. Dr. A.'s classical education was, confessedly, neglected, and he had made no preparation for the examination, when he was suddenly called apon to undergo the trial, in consequence of the canvass for the Fever Hospital. We know that his pulse was 130 when he presented himself in Warwick-lane, having been exhausted by fatigue and heat that day. He was, in fact, in a nervous fever at the time, and we have no doubt that he stumbled in the majority of questions put to him by the learned body at the Censor's Board. The writer of this article was examined on the same day when the late Dr. Good was rejected. With not a tenth of the medical learning possessed by that distinguished author, he easily passed the Board by maintaining tranquillity of mind, and by not embarrassing himself with attempts to clothe the A. B. C.'s of physic in fine Latin. The examples of Drs. Armstrong and Good, and the impression which their rejection by the College made on the public, have been serviceable to the Board of Censors ever since. They have lowered their tone, and taken care not to trifle with the feelings of the community by similar expositions of the bad system of examination to which they are tied down by monkish regulations.

In 1821, the chief engine of Dr. Armstrong's success was put in play, by the commencement of his lectures in Maze Pond, at Mr. Grainger's theatre. The impassioned manner in which Dr. A. lectured, and the flow of language which was at his command, rendered him extremely popular, and insured him success. In 1826, he opened another school in Little Dean Street, Soho; but it must be confessed that this was a complete failure, though his biographer attributes the abandoning of the scheme to want of time and health. We have no doubt, indeed, that his exertions at this period undermined his health. His ambition was boundless, and he grasped at more than human nature, or, at all events, his own constitution, could possibly accomplish. We have reason to believe that, in 1828, he was in the receipt of from three to four thousand pounds per annum. Fame magnified this to double its amount; but we venture to assert that we have placed his practice at the highest pitch which it ever attained. And truly it was a splendid point to attain in the course of ten years residence in the metropolis, where men of the highest talents and acquirements too often live and die, without ever arriving at half such an income from their practice.

Dr. Boott has omitted many particulars in the life of his favourite preceptor, which are familiar even to his common acquaintances. It is well known that Dr. Armstrong always had a hankering for the west end of the town. His usual observation was, that although he rose in the East he should like to set in the West. He had all but determined on taking a house in Cavendish Square a few years before his death. Though no admirer of courts or palaces, Dr. A. had probably a wish to enlarge, or even to change, the class of patients among whom he chiefly practised. Russell Square, the terra incognita, as Theodore Hook pronounced it, is neither City nor West End, but almost entirely composed of legal and mercantile inhabitants. It is very likely that the ardent and imaginative mind of Dr. Armstrong longed for change of scene and new sources of excitement. The projection of the Dean-street school was, perhaps, a part of the plan of migration, which, however, was never put into execution.

It was in the Winter of this year (Dec. 1828) that Dr. A. manifested symptoms of fixed and serious disease. He had cough, which he attributed to public speaking; and his bodily debility he attributed to fatigue. Before he left Sunderland, however, he was affected with a cough, which created some apprehension even in his own mind, as was evident from certain expressions in a letter to the Editor of this Journal in 1817. Dr. A. was obliged to interrupt his Autumnal course this year, for a few weeks, and go into the country, but recommenced in January, 1829. From this period to the day of his death, the struggle between energy of mind and disease of body was most melancholy to behold. It is to be regretted that neither Dr. A., nor any of his friends, entertained suspicions of his health till a fatal malady had taken deep root. But even when the alarm was universal in the family and friends, the patient's obstinacy baffled every means of arresting the progress of the disease. "He was at all times impatient of advice, and would seldom permit his medical friends to counsel him." Thus, in the middle of February, 1829, he was obliged to suspend his lectures, and was prevailed on to go to Seven Oaks, in Kent, where he obtained immediate benefit, his cough almost leaving him, and refreshing sleep having returned. But the moment he got a little better he returned to the oar, and worked like a galley-slave, till another fit of exhaustion deprived him of physical power to proceed. On the 3d of March (29), he had the imprudence to dine with the Webb-street pupils at the Freemason's Tavern. In the beginning of May he was forced to retire to Burford Bridge, near Dorking, and there, for the first time, he expressed apprehension that he laboured under phthisis. Yet, on the 17th of the same month, he returned to town—recommenced practice—"and said he had found all his unfavourable symptoms rapidly decline." He soon again relapsed into debility, became very irritable, "and seemed at times to look hopelessly around for relief." Early in June, Dr. Boott entreated him to leave off lecturing for several months, and seclude himself in the country; but he clung so much to the practice of his profession, that he would not recede out of the reach of London daily. However, he was soon obliged to secede from the task of delivering the Summer course of lectures, and compelled Dr. Boott to undertake the formidable post of substitute. Dr. A. now went to Worthing, with his family. There he seemed to have got a sudden accession of strength, so as to be able to walk several miles along the beach on the evening of his arrival. The beach, therefore, became his favourite resort, and he often sought to cool his hectic fever by the refreshing sea-breeze. He was now sanguine of recovery, and even his friends indulged the same fond hope. The improvement was but transient.

"The improvement in his health and spirits was but transient; yet the good effects he had so uniformly though transiently experienced from change of scene, led him to indulge in sanguine hopes of the future, and to take at times a different view of the nature of his disorder from that which he expressed to me on the morning of his leaving town for Box Hill. He would seldom admit to those about him that there was anything serious in his case, and he seemed occasionally to have almost convinced himself that he laboured under chronic pleurisy. He complained of the excitement which the visit of his medical friends produced, and would not allow them to see him. When pressed upon this point, he constantly urged the necessity of complete seclusion, so that I had no opportunity of seeing him for several weeks after he left London." 79.

At length Dr. A. desired to see Dr. Boott, and the latter repaired to Worthing in conjunction with Dr. James Clark. They found Dr. A. so weak as to be unable to walk across the room. He lay on the sofa, with his watch frequently in his hand, counting his pulse. Dr. C. considered that there were no hopes left. His cheerfulness, however, had not entirely deserted him, and he sometimes rallied his friends around him on their sombre looks. They left Worthing, and shortly afterwards Dr. B. received a letter from Mr. Spearman, Dr. Armstrong's brother-in-law, from which we shall quote a remarkable passage.

"His night perspirations have left him—his fits of excitement are very much abated—his cough less frequent, and we all think he improves in appearance and flesh. He is also much more comfortable and happy in mind, though I know not whether to think the improvement of his spirits a favourable symptom as regards his disorder, of which he speaks in more sanguine terms than he has done since he came here. He told me, after you were gone, that he was quite satisfied both yourself and Clark thought his case hopeless, and that he saw through your evasions of his questions on this point, but that such a conclusion was by no means warranted by the symptoms and circumstances of it. In short, he seems determined to recover, in order to confute you both. Pray God he may! but I know not whether we may rationally indulge a hope." 82.

On the 4th August, (1829,) Dr. A. left Worthing, dissatisfied with the air of the place, and came once more to Burford Bridge, where he again seemed rapidly to improve under the influence of the genial atmosphere of the place. From this place Dr. Boott received a letter prohibiting a visit from his medical friends.

"'I shall say something about your health, connected with lecturing, when I see you. But you and Clark must defer your visit for two or three weeks, for that at Worthing shook me dreadfully, and I have not yet fully recovered from the depression and collapse induced by Clark's visit the other night. Thus my extreme excitability converts the kindness of my friends into a cause of aggravating my malady. If I recover, I shall give you and him a lecture for your want of *tact*, in not having taken this excitability into account. When I get over my depression and collapse completely,—and they are much lessened to-day,—I shall be better, I trust. Pray tell Clark to defer his visit awhile. But I have been scolding you when I should say 'God bless you.'" 84.

He began to take exercise in the open air, and passed the greater part of the day in walking or riding. On the 3rd September, he wrote to Dr. B. that, on the whole, he was better, and determined to be well in five weeks. On the 15th September Dr. B. ventured to go down to see him. Dr. B. was then convinced that no essential change for the better had taken place. Yet he was able to take a long walk, though at Worthing he could scarcely stand on his feet. He had adopted a more generous diet, and took ale. Yet he had a presentiment of the fatality of his disease, on account of a symptom which caused great alarm. This symptom was more explicitly adverted to on the 25th of the same month.

"After dinner he took me to the beautiful grounds of Mr. Denison, talking all the time we ascended the hill; and in the evening, till as late as eleven o'clock, we walked to Leatherhead and back. On our return we observed some glow-worms on a bank by the road-side, and he caught several and desired I would take them to the children. He did not complain of cold, though the night air was chill, and a dense fog hung over the bottom of the valley. I repeatedly urged him to shorten our walk; but he remarked that he had generally been out as late, and that he slept the better for it. He again alluded to the symptom of which he spoke before on my last visit, and said that it was a death-watch forewarning him of his doom. This was the *metallic tinkling* of Laennec, which he compared to the faint vibrations of air, caused by the gentle stroke of the hammer of a silver bell. He spoke of it as always present to his ear, and that it was a fatal indication." 89.

This is a very curious circumstance. There was a large excavation in the left lung, it is true; but this was not a case, according to Laennec, where such a phenomenon as the tintement metallique should have presented itself. We are confident, indeed, that the phenomenon has not yet been clearly accounted for, and that it may be present in different and almost opposite conditions.

While the above subject was present to his mind, he spoke of his death as inevitable; yet, the very next day, we find Dr. A. forming the plan of a journey to Durham, and looking forward with pleasure to his return to practice in London! On the 2d October he came to town, apparently much better, and with powers of body greatly renovated, though the emaciation continued. These and other circumstances render it probable, that a secession from London turmoil, and a tour of health for five or six months, would have saved this valuable life! On the 8th October, he left town for Durham. He travelled in an open carriage, and arrived in Durham on the third day, without fatigue; but he soon had a relapse there, and his visit to the place of his nativity did him no good. He returned to London on the first of November.

"It was most affecting to witness the persevering but ineffectual efforts he made to rally his sinking energies, and to enter once more upon practice. His mind seemed to infuse vigour into his wasted and enfeebled frame, and for two or three weeks he was much occupied in visiting patients, in and for a distance of several miles from town. But he came home always in a state of great exhaustion, and it was painful to observe his instinctive promptness to attend to the calls of duty, blended with the incapacity of exerting himself without greatly aggravating his sufferings. He could not be persuaded to abandon all thoughts of his profession; and it seemed a relief to him to feel that he was yet capable in some degree of being useful to his family." 92.

On the 19th, Dr. B. was hastily summoned, and found his afflicted friend in great distress, under the idea that one of his ribs was fractured, and that this had all along been the cause of his illness. The suspicion was entirely groundless. He soon after this took to his bed, never more to rise from it. On the 7th Dec. a consultation took place, and Dr. Davies, of Broad-street, examined with the stethoscope—far too late. An excavation was found in the upper part of the left lung. On the 12th Dec. our author was called to him.

"About four o'clock he called me, and repeated the summons before I could reach his bed-side; and when I did so, he said, 'I am too impatient, and grieve to give so much trouble. In writing my Life, which can only be useful as a history of my opinions, enter into no controversy about me, or my cause of offence against the College. And if you are censured, say it was my last request, that I might die in the consciousness of being at peace in the grave. I die in peace with all mankind.'" 99.

He died that night. The following are the minutes of the post-mortem examination.

"The body presented the appearance of extreme emaciation: the adipose substance was totally absorbed, the muscles unusually red, and their texture of moderate firmness.

On opening the chest, the upper third of the left lung was seen firmly adhering anteriorly to the costal pleura; a large tubercular excavation corresponded in extent to that portion of the lung, capable of containing from twelve to sixteen ounces of fluid. The whole of the anterior parietes of this cavity were not above two or three lines in thickness, formed of condensed pulmonary structure, and firmly adherent in the whole of its extent to the pleura costalis. The posterior parietes of the excavation were considerably thicker, and traversed by a number of bands of pulmonary structure, studded with tubercles, constituting what are called '*columns*' by Bayle. The cavity was partially lined with a false membrane of various consistency, in some parts soft and easily scraped off with the scalpel, in others of a cartilaginous density. Various bronchial openings existed in the right and upper portion of the excavation. The remaining portion of the left lung was filled with tubercles in all their stages,—the grey, the crude, the softening.

The upper half of the right lung was filled with tubercles accumulated in rounded masses, the interval between these masses being tolerably healthy. There was also in the apex of the same lung an excavation capable of holding a small-sized walnut. This was lined with a firm membrane. The inferior half of the right lung presented a few disseminated tubercles, but its structure was not materially different from the healthy state.

There were but few adhesions; the heart was small, its texture soft, but not diseased.

The direction of the stomach was more perpendicular than usual; its mucous membrane was generally softened and reddened.

The intestines were healthy, except near the ileo-colic valve, where a mass of the glandulæ aggregatæ were in a state of hypertrophy.

The rest of the viscera were in their natural state, with the exception of the gall-bladder, which contained a stone of an oval form, about the size of a hazel-nut.

Thomas Davies, M.D.
George Langstaff.
George Pilcher.
Francis Boott, M.D." 103.

This talented physician was in the 46th year of his age, when death deprived the world of his talents and his family of their supporter! He left six children—three sons and three daughters. From Dr. Boott's portrait of the author we select the more important features.

"In person Dr. Armstrong was tall and thin. His manners were gentle and unpresuming, almost diffident in the presence of strangers, exclusively domestic and retired from the world, when the calls of duty did not require his intercourse with it. His nature was candid, confiding, unsuspicious; his sensibilities lively and acute; his tastes discriminating and refined. There was a simplicity and innocence of mind and disposition about him which endeared him to all who knew him intimately, and which won for him especially the confidence and attachment of the young." 104.

"He carried the sagacity which he displayed in his profession into his observations of life, and none of its more delicate and evanescent beauties seemed to escape his notice. It formed one of the peculiar charms of his conversation to observe from it how quickly and sensitively he perceived and felt those instinctive actions and expressions which give individuality to character." 105.

"Though susceptible of a playful humour and an occasional gaiety of feeling, he was naturally of a serious cast of character; and from the absorbing interest he took in his profession, the distressing scenes he moved in, and the thoughtful bent of his mind, he often wore a look of abstraction and concern. He was wholly unsuited to mix in the pursuits of fashionable life. He had no community of taste with its admirers, nor capacity to appreciate the trifling objects of momentary interest which the fluctuating surface of society presents to those who are content with its fleeting novelties. His only sources of happiness were in the contemplation and practice of his profession, in the hallowed seclusion of home, and in the society of a few intimate friends.

He was more exclusively and anxiously devoted to the duties of his profession than any man I ever knew. Nothing it required ever appeared to him an encroachment upon his time, or an invasion of his ease. He never refused to attend to the calls of distress, and was always most liberal of his time and advice to the poor.

His pupils were warmly attached to him, from the interest he took in their improvement and their comforts. He watched over them in sickness with paternal solicitude. He blended so much of the generosity of his nature, the sensibility of his feelings, and the purity of his tastes in his lectures, that no public teacher was ever held in higher admiration or respect. No one who was unable to incur the expense of taking his tickets was ever dismissed as an unsuccessful applicant. Such, indeed, always found in him the friend in need; for he obtained for them free admission to the lectures of his colleagues, and assisted them in other ways to complete their medical education." 107.

Dr. Armstrong was fond of poetry, and Dr. B. has introduced several specimens of his honoured preceptor's composition. We select the last, because it is the shortest as well as the best. It was written in 1808.

"THE WISH.

Obscurely let me live and die—
But not unknown, unhonour'd lie;
Oh! may the poor and wretched trace
My grave in some sequester'd place,
Where nameless streams my requiem sound,
And nature breathes in beauty round.
There, ardent, may their blessings rise,
Like incense offered to the skies." 110.

The biographer concludes, as we before mentioned, by asserting that he could perceive no defects in the moral character of Dr. Armstrong. We doubt the propriety of this declaration, as it savours more of romance than real history. No man was ever without defects both in mental and physical constitution. To conceal or overlook the foibles of our friends, in biographical delineations, is not profitable to the living. It is like publishing all our successful cases, and keeping the unsuccessful ones in the back ground. We who have uniformly upheld the character and praised the writings of Dr. Armstrong, during life, need not dread the charge of envy, hatred, or malice, if we acknowledge that our friend and favourite author was not exempt from defects, no more than his fellow-creatures. But we refer our readers to our Journal for January 1830, where we have given a full and a candid estimate of Dr. Armstrong's character, and where we have pointed out what we conceive to have been defects in that talented physician. Dr. Boott has executed his melancholy task with all the good feeling of an admiring pupil delineating the character of an almost adored preceptor. If, in the performance of this difficult duty to the dead and the living, Dr. Boott has been unwilling or unable to discover any weak point in his favourite tutor, the failure is at least amiable, if not praiseworthy, and we hereby tender him the homage of our respect.

The greater part of the volume is occupied with two subjects—one, a kind of review or exposition of Dr. Armstrong's opinions respecting fever—and the other, a long and laborious survey of the fevers which prevail in the United States, from the latitude of 19° to that of 42°—viz. from Vera Cruz to Boston. In all the fevers and types of fever, including yellow fever, typhus, bilious remittents, and intermittents, the Doctor recognizes but one general remote cause—malaria, whether the result be marsh effluvium or animal and vegetable decomposition. The various fevers themselves, he considers as only modifications of the same disease, dependent on the climate of the place, and the greater or less intensity of the cause. He proscribes contagion from the agents that produce it—at least that high grade of it denominated yellow fever.

We doubt whether we should be able to give a satisfactory analysis of this, the greater part of the work, being itself indeed a review. Nevertheless we shall endeavour, if possible, to present our readers with a succinct view of that portion in our next number. Meantime we recommend the work to the public in general, and to the numerous pupils and admirers of the late lamented Dr. Armstrong in particular.

